# Mechanism of crocin I on ANIT-induced intrahepatic cholestasis by combined metabolomics and transcriptomics

**DOI:** 10.3389/fphar.2022.1088750

**Published:** 2023-01-18

**Authors:** Dandan Song, Pei Zhu, Yankai Dong, Mengchao Wang, Anna Zhao, Hongdong Xia, Yunting Chen, Qingguang Zhou, Lun Xiang, Junyi Zhang, Guangming Luo, Yangjing Luo

**Affiliations:** ^1^ Jiangxi University of Chinese Medicine, Nanchang, Jiangxi, China; ^2^ Northwest University, Xi’an, Shaanxi, China; ^3^ Nanchang University, Nanchang, Jiangxi, China

**Keywords:** crocin I, intrahepatic cholestasis, metabolomics, transcriptomics, comprehensive strategy

## Abstract

**Background:** Intrahepatic cholestasis (IC) is a disorder of bile production, secretion, and excretion with various causes. Crocin I (CR) is effective in the treatment of IC, but its underlying mechanisms need to be further explored. We aimed to reveal the therapeutic mechanism of crocin I for IC by combining an integrated strategy of metabolomics and transcriptomics.

**Methods:** The hepatoprotective effect of CR against cholestasis liver injury induced by α-naphthylisothiocyanate (ANIT) was evaluated in rats. The serum biochemical indices, including alanine aminotransferase (ALT), aspartate aminotransferase (AST), total bile acid (TBA), total bilirubin (TBIL), direct bilirubin (DBIL), tumor necrosis factor-α (TNF-α), interleukin 6 (IL-6), and interleukin 1β (IL-1β), as well as the liver oxidative stress indexes and the pathological characteristics of the liver were analyzed. In addition, we also performed a serum metabolomics study using UPLC-Q Exactive HF-X technology to investigate the effect of CR on the serum of rats with ANIT-induced IC and screened potential biomarkers. The enrichment analysis of differential expressed genes (DEGs) was performed by transcriptomics. Finally, the regulatory targets of CR on potential biomarkers were obtained by combined analysis, and the relevant key targets were verified by western blotting.

**Results:** CR improved serum and liver homogenate indexes and alleviated liver histological injury. Compared with ANIT group, the CR group had 76 differential metabolites, and 10 metabolic pathways were enriched. There were 473 DEGs significantly changed after CR treatment, most of which were enriched in the retinol metabolism, calcium signaling pathway, PPAR signaling pathway, circadian rhythm, chemokine signaling pathway, arachidonic acid metabolism, bile secretion, primary bile acid biosynthesis, and other pathways. By constructing the “compound-reaction-enzyme-gene” interaction network, three potential key-target regulation biomarkers were obtained, including 3-hydroxy-3-methylglutaryl-coenzyme A reductase (HMGCR), ATP-binding cassette transporter G5 (ABCG5), and sulfotransferase2A1(SULT2A1), which were further verified by western blotting. Compared with the ANIT group, the CR group significantly increased the expression of ABCG5 and SULT2A1, and the expression of HMGCR significantly decreased.

**Conclusion:** Combined metabolomic and transcriptomic analyses show that CR has a therapeutic effect on IC through regulation of the biosynthesis of bile acids and bilirubin in the bile secretion pathway and regulation of the expression of HMGCR, ABCG5, and SULT2A1.

## 1 Introduction

A common type of liver disease, intrahepatic cholestasis (IC) is a primary hepatocellular disease. Bile formation and blood flow are blocked due to damage to hepatocytes and bile duct cells. In particular, bile acids are trapped, and bile components such as cholesterol and bilirubin continue to accumulate (Yang et al., 2018). Disturbances in bile acid metabolism and inflammation are common features of IC, with clinical symptoms of jaundice, itchy skin, darkened urine, and physical frailty. If the cholestatic state is not effectively treated, it will develop into liver fibrosis, and even liver cirrhosis (Zhang et al., 2022). Ursodeoxycholic acid, obeticholic acid, S-adenosylmethionine, and other drugs are often used for the clinical treatment of intrahepatic cholestasis. However, the use of ursodeoxycholic acid will cause intolerance in patients. Obecholic acid can produce side effects such as itching (Samant et al., 2019; Fujita et al., 2021). Therefore, the search for an effective drug with few side effects to treat IC is an important topic. Chinese medicines and their monomers have the advantage of being widely applied and have few side effects. Terpenoids have various anti-inflammatory, antioxidant, and anti-fibrosis pharmacological activities that can effectively alleviate IC, and terpenoids are expected to become new drugs for the treatment of cholestatic liver disease (Ji et al., 2022).

**FIGURE 1:**
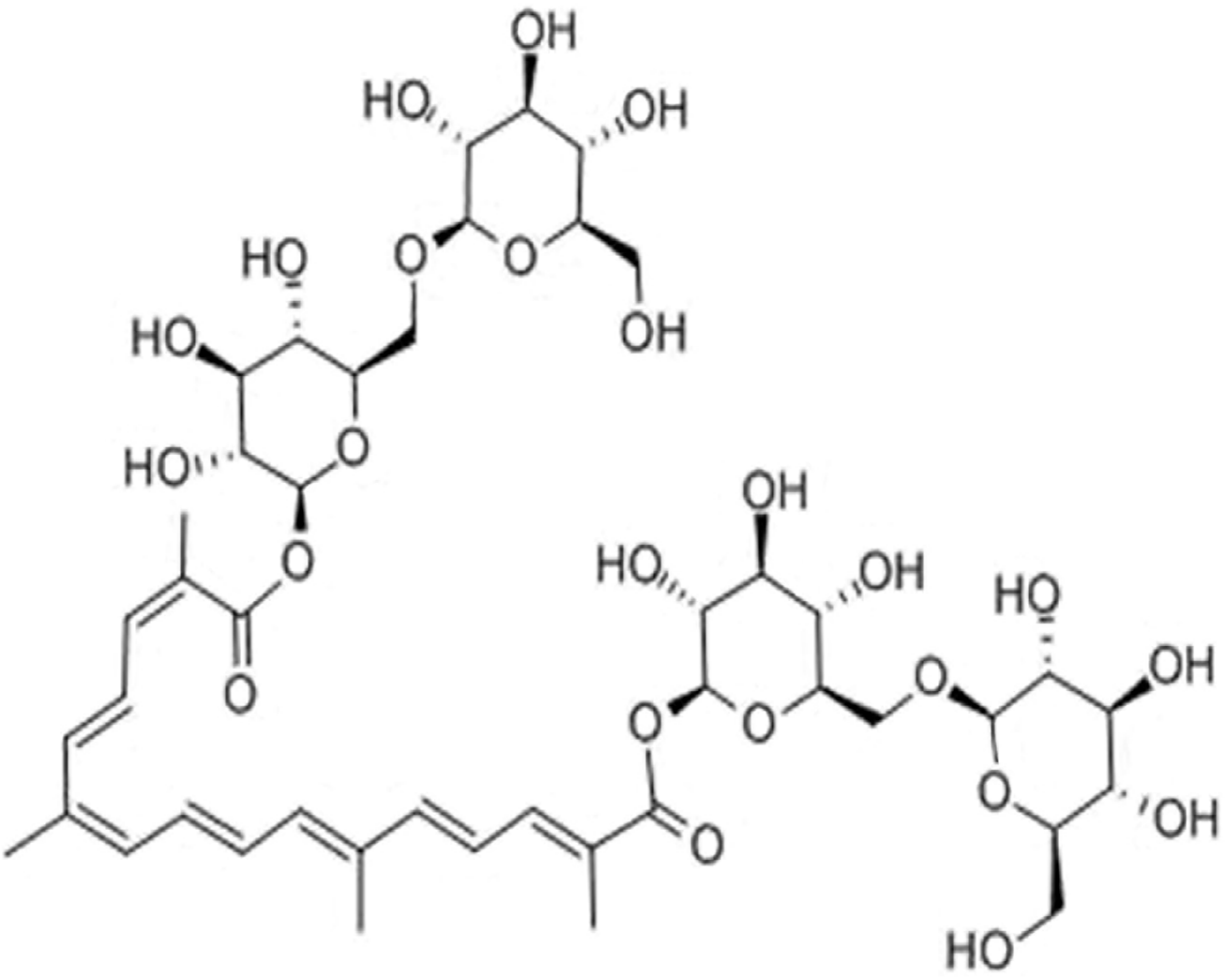
The structure of crocin I.

Crocin I (CR), as a diterpenoid, is the main active component of saffron (Figure 1). In recent years, many studies have shown that CR has hepatoprotection (Lari et al., 2015), antitumor (Tang et al., 2022), anticardiovascular disease (Motlagh et al., 2021), anti-inflammatory (Teng et al., 2021), and hypoglycemic (Qiu et al., 2020) effects. In addition, it has less toxicity and fewer side effects, and its medical value has received increasing attention. However, the mechanism of how CR alleviates IC is still unclear. Combining the relevant gene expression information provided by transcriptomics with the statistical information of metabolomics data variables is a reliable method of studying the material basis and mechanism of action of the pharmacological effects of Chinese medicine monomers. Currently, integrating the two techniques to explore the mechanism of CR in the treatment of IC remains a void.

Metabolomics is an important part of systems biology. By revealing the metabolic trends and laws of the body under the influence of internal and external factors, metabolomics can qualitatively and quantitatively determine the dynamic changes of metabolites in the living system caused by pathological, physiological stimulation, or genetic modification (Alarcon-Barrera et al., 2022). The mechanism of how CR protects the liver can be elucidated through metabolomics. Genes are closely related to changes in metabolites, so transcriptomics is used to explain changes in gene expression after drug administration. In addition, combined metabolomic and transcriptomic analysis can reveal phenotype-related metabolic pathways and gene functions through the identified metabolites and differential genes to further gain potential therapeutic targets for various types of liver injury and elucidate disease pathogenesis.

**FIGURE 2:**
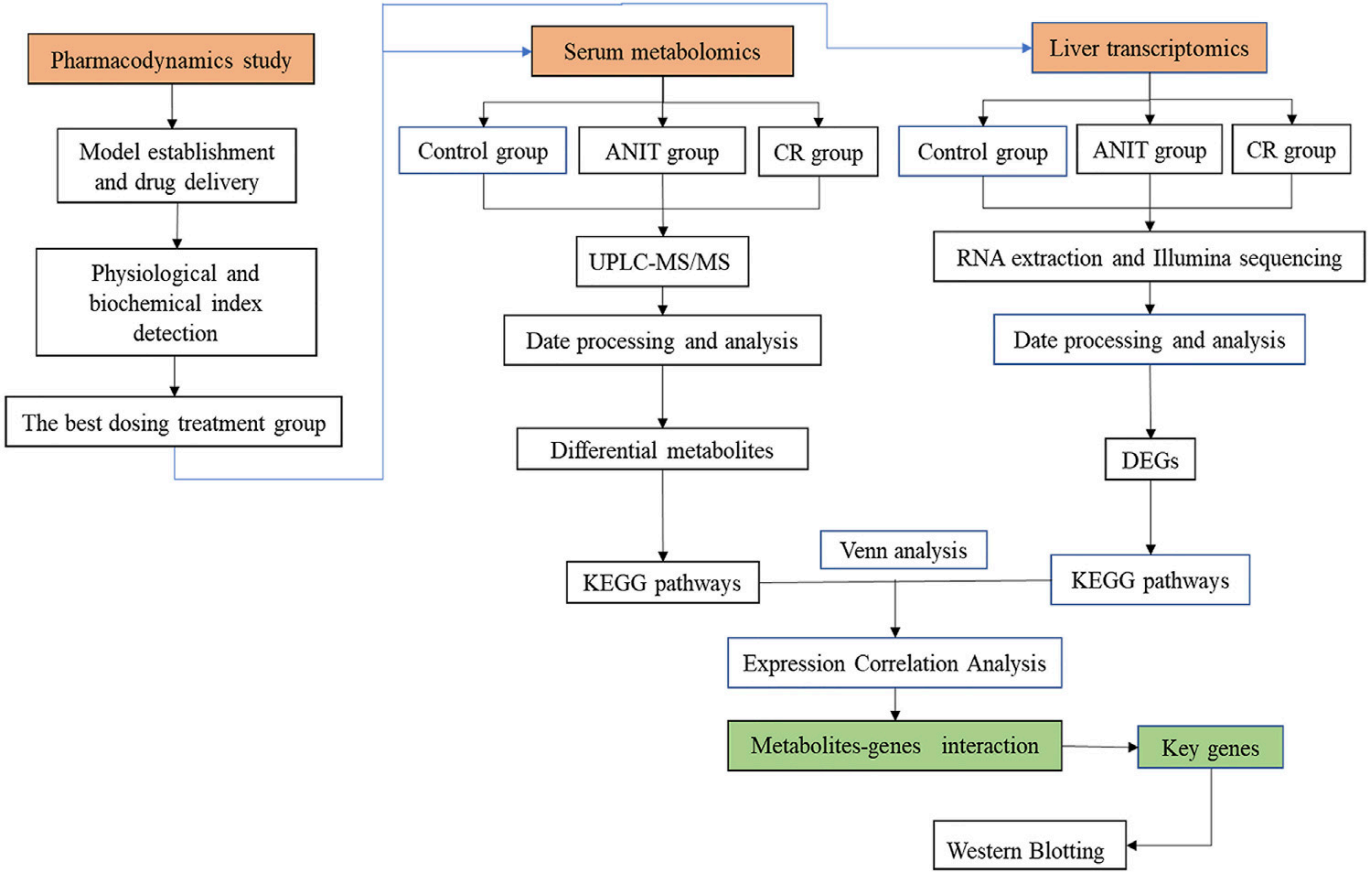
The schematic flowchart of the integrated strategy.

In this study, metabolomics and transcriptomics were integrated. The effects of treating IC with CR on essential metabolites were determined with untargeted metabolomics. We then developed a novel integrated strategy to explore the key targets and mechanisms of CR in treating acute IC based on metabolomics and transcriptomics. This study provided new insights into the protective effects of CR in the treatment of IC. The research flowchart is shown in Figure 2.

## 2 Materials and methods

### 2.1 Chemicals and reagents

Crocin I (C44H64O24, purity>98%, BCTG-0315) was purchased from the National Engineering Research Center of Traditional Chinese Medicine Solid Preparation Manufacturing Technology (Nanchang, China). ANIT and ursodeoxycholic acid (UDCA) were purchased from Shanghai McLean Biochemical Technology Co., Ltd. (Shanghai, China). The alanine aminotransferase (ALT), aspartate transaminase (AST), total bile acid (TBA), total bilirubin (TBIL), and direct bilirubin (DBIL) kits were purchased from Nanjing Jiancheng Bioengineering Institute (Nanjing, China). The tumor necrosis factor-α (TNF-α), interleukin-6 (IL-6), interleukin-1β (IL-1β) kits were purchased from Xinbosheng Biotechnology Co., Ltd. (Shenzhen, China).

### 2.2 Animals

Male Sprague–Dawley rats weighing 200 ± 20 g were obtained from Huiji District (Zhengzhou) Experimental Animal Farm (Zhengzhou, China, Permission No. SCXK(Yu)2019–0002). All rats were housed in a well-ventilated room at 25°C, 60% humidity with a 12-h dark-light cycle and free access to food and water. Animal experiments were carried out in accordance with the guidelines for animal experiments at Jiangxi University of Traditional Chinese Medicine.

After all the rats were allowed to acclimate for 1 week, 48 rats were randomly divided into six groups (n = 8/group): CON group, ANIT group, UDCA group, CR-L group, CR-M group, and CR-H group. The CR groups and the UDCA group were given corresponding drugs by prophylactic gavage. The UDCA group received 60 mg/kg (Li et al., 2020), the CR-L group received 10 mg/kg, the CR-M group received 30 mg/kg, and the CR-H received 90 mg/kg for seven consecutive days, once a day. The CON group and the ANIT group were given an equal volume of .5% CMC-Na. Two hours after administration on the fifth day, the CON group received olive oil, and other groups were given 75 mg/kg ANIT olive oil, respectively.

### 2.3 Sample collection and preparation

After anesthesia with 3% pentobarbital sodium, blood samples were taken from the abdominal aorta and liver. Next, the blood was centrifuged at 3,000 rpm for 10 min to separate the serum and transferred to −80°C for preservation. Then, the relevant indexes were measured using the appropriate kits. The changes in the physiological and biochemical indexes of rats after CR treatment were observed through histopathological and biochemical analysis.

### 2.4 Histological examination

The liver tissue was fixed with 4% tissue cell fixative solution. The fixed liver tissue was taken out for routine dehydration, paraffin embedding, sectioning, HE staining, microscopy, and image acquisition.

### 2.5 Metabolomics analysis based on UPLC-Q-Exactive HF-X MS

#### 2.5.1 Sample collection and preparation

According to the results of 3.1, the CON group, the ANTI group, and the CR-H group were selected for follow-up analysis of serum. A 100 µL aliquot of the sample was precisely pipetted into a 1.5 mL centrifuge tube, and 400 µL of extraction solution was added (methanol: acetonitrile = 1:1 (v:v)), containing .02 mg/mL internal standard (L-2-chlorophenylalanine). After vortexing for 30 s, low-temperature ultrasonic extraction was performed for 30 min (5 °C, 40 KHz). The sample was placed at −20°C for 30 min and centrifuged for 15 min (13000g, 4°C). The supernatant was removed, and was blown dry with nitrogen; 100 μL of the reconstituted solution was added (acetonitrile: water = 1:1) for reconstitution, vortexed for 30 s, extracted by low-temperature ultrasonic extraction for 5 min (5°C, 40 KHz), and centrifuged for 10 min (13,000 g, 4°C). The supernatant was transferred to a sample vial with an inner cannula for analysis on the computer. The supernatant was stored at −20°C for LC-MS analysis.

#### 2.5.2 Chromatography and mass spectrometry

Serum samples were analyzed using a Thermo Fisher Scientific UPLC-Q Exactive HF-X system. The chromatographic column was ACQUITY UPLC HSS T3 (100 mm × 2.1 mm id, 1.8 µm; Waters, Milford, USA), and the column temperature was 40°C. The mobile phase A was 95% water and 5% acetonitrile (containing .1% formic acid). Phase B was 47.5% acetonitrile, 47.5% isopropanol, and 5% water (containing .1% formic acid), and the injection volume was 2 μL.

In positive and negative ion mode, the linear gradient of the elution column is 0–3.5 min, 0%–24.5% B; 3.5–5 min, 24.5%–65% B; 5–5.5 min, 65%–100% B; 5.5–7.4 min, 100% B; 7.4–7.6 min, 100%–51.5% B; 7.6–7.8min, 51.5%–0% B; 7.8–10min, 0% B. The flow rate was 0.4 mL/min. The scanning range (m/z) was 70–1050 Da; the sheath gas flow rate was 50 arb; the auxiliary gas flow rate was 13 arb, the spray voltage (positive and negative mode) was 3500V, −3500V, the heating temperature was 425°C; the capillary temperature was 325°C, and the collision energy was 20, 40, 60 eV.

#### 2.5.3 Method validation

A 20 µL aliquot of the supernatant was pipetted from each sample and mixed as a quality control sample (QC). The volume of each QC was the same as that of the sample, and it was processed and detected in the same way as the analytical sample. During the instrument analysis process, a QC sample was inserted after every 10 analysis samples to examine the stability of the entire detection process.

#### 2.5.4 Data preprocessing and differential metabolite analysis

The raw data were imported into the metabolomics processing software Progenesis QI (Waters Corporation, Milford, USA) for baseline filtering, peak identification, integration, retention time correction, and peak alignment*.* A data matrix containing information such as retention time, mass charge ratio, and peak intensity was obtained (Trezzi et al., 2015; Vignoli et al., 2020). The R software package ropls (Version 1.6.2) was used to perform multivariate statistical analysis of PLS-DA and OPLS-DA on the processed data. VIP>1 indicated that the metabolites had an important effect on the classification between groups. The model was tested for overfitting by permuting 200 times. The MS and MS/MS mass spectral information was matched and identified using the HMDB (http://www.hmdb.ca/) database and the Metlin (https://metlin.scripps.edu/) database to meet the VIP >1 and *p* < .05 differential metabolites through metabolic pathway enrichment analysis on data by the KEGG database and SciPy (Python) (Chong & Xia, 2018).

#### 2.5.5 RNA extraction and sequencing

The liver tissue was taken from the −80 ° refrigerator, and the total RNA was extracted by the TRIzol (Invitrogen) method. The sequencing experiment was performed using the Illumina TruseqTM RNA sample prep kit method for library construction. Seqprep and Sickle software programs were used for preprocessing to obtain clean data. The data after quality control were compared with the published rat genome sequence for *Rattus norvegicus*. The DEGseq2 software was used to analyze the differential expression of the CON group, the ANIT group, and the CR-H group, and the differentially expressed genes (DEGs) were screened. Then, the GO and KEGG databases were used for the enrichment analysis of the DEGs.

#### 2.5.6 Western blotting

Three samples from each group were selected for western blot analysis and comparison. The total protein of the samples was extracted with RIPA tissue cell rapid lysis buffer, centrifuged at 12000g at 4°C for 15 min, and the supernatant was collected for protein quantification and stored in a −80°C refrigerator. Then, the BCA kit was used to measure the protein concentration. The protein was separated with 10% SDS polyacrylamide gel and transferred to a polyvinylidene fluoride PVDF membrane. The PVDF membrane was blocked with 5% nonfat milk powder at room temperature for 1 h or overnight at 4°C. Afterward, it was washed with TBST, the primary antibody was incubated at 4°C overnight, and the membrane was incubated with the primary antibody three times, 5 min each time. Then, according to the dosage, the HRP-labeled secondary antibody was diluted 1:1000 and incubated with the membrane at 37°C for 1 h. TBST was used to wash three times for 5 min each. Finally, ECL chemiluminescence detection was performed for development. GAPDH was selected as the internal reference, and Image J software was used for gray value analysis.

### 2.5.7 Statistical analysis

Statistical analysis of the data was performed using Graphpad Prism 8.0 software, and the results were expressed as mean ± standard deviation (X ± S). One-way ANOVA was used for statistical differences among each group, and Scheffe was used for the *post hoc* test for comparing every two groups; *p* < .05 indicated statistical significance, and *p* < .01 indicated extremely significant differences.

## 3 Results

### 3.1 Effects of CR on ANIT-induced IC in rats

Biochemical and histological analyses were used to evaluate the pharmacodynamics of CR in the treatment of IC.

**FIGURE 3:**
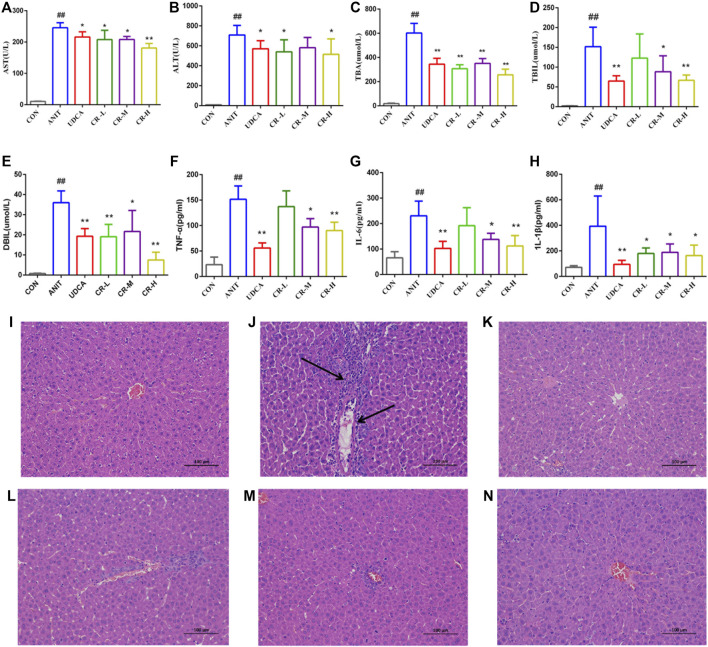
Effect of crocin I on serum biochemical indexes and histological (H&E stained, 100× magnification). Hepatocyte damage is indicated by black arrows. **(A)** Serum AST level, **(B)** serum ALT level, **(C)** serum TBA level, **(D)** serum TBIL level, **(E)** serum DBIL level, **(F)** serum TNF-α level, **(G)** serum IL-6 level, **(H)** serum IL-1β level, **(I)** CON group, **(J)** ANIT group, **(K)** UDCA group, **(L)** CR-L group, **(M)** CR-M group, **(N)** CR-H group. Data are presented as means ± SD (n = 6). #*p* < .05, ##*p* < .01 compared with the CON group; *p < .05, **p < .01 compared with ANIT group.

As shown in Figures 3A–H, compared with the CON group, the levels of AST, ALT, TBA, TBIL, and DBIL in the serum of the ANIT group significantly increased, indicating that the ANIT group had a severe liver injury. Compared with the ANIT group, the middle- and high-dose CR and UDCA groups showed significantly reduced levels of AST, ALT, TBA, TBIL, DBIL, IL-Iβ, IL-6, and TNF-α (*p* < .05). The CR-H group showed the most significant effect (*p* < .01).

The histological evaluation provided more intuitive evidence for the protective effect of CR on ANIT-induced IC. As shown in Figure 3I-N, the liver tissue of the CON group showed a standard cellular structure, and the ANIT group showed typical pathological changes, including inflammatory infiltration and necrosis of liver cells and a small amount of hepatic fibrosis and connective tissue proliferation. The degeneration of hepatocytes in the UDCA group and the CR-L, CR-M, and CR-H groups was alleviated, the infiltration of neutrophils was less, and the CR-H group had the most obvious therapeutic effect, which indicated that CR had a certain protective effect on IC.

### 3.2 Multivariate statistical analysis

**FIGURE 4:**
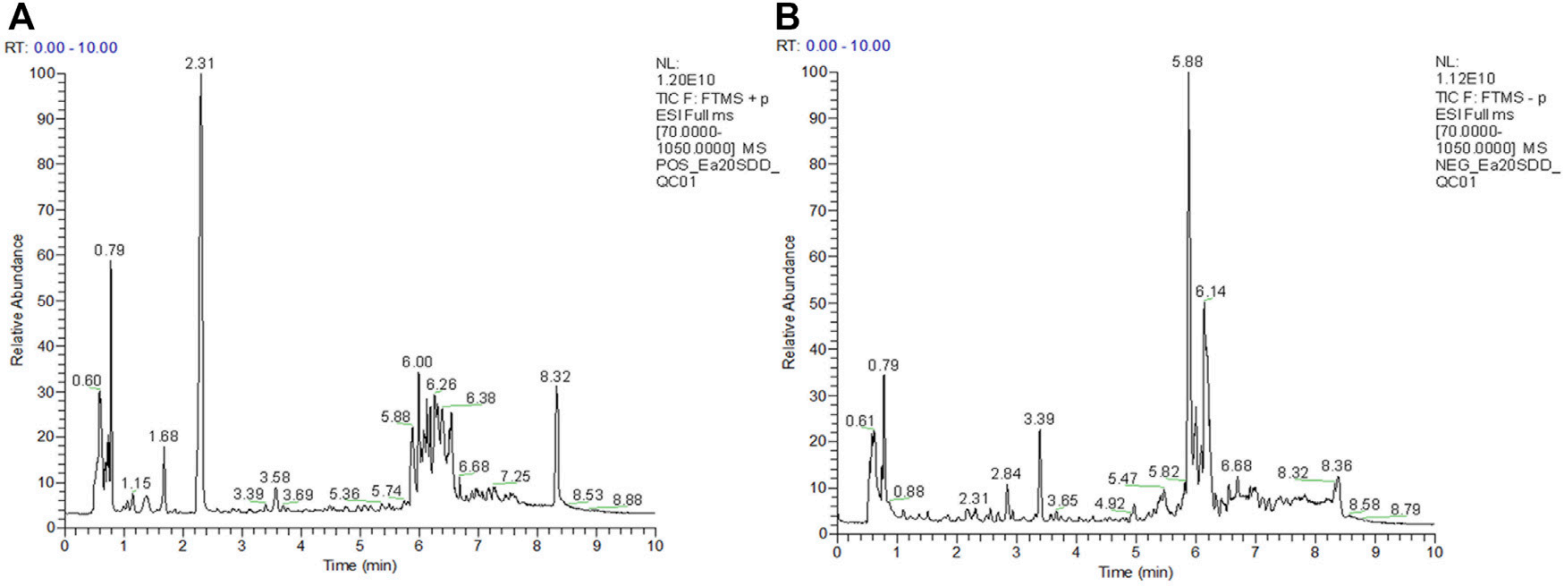
Typical chromatograms of positive and negative modes. **(A)** UPLC-QE ESI(+) total ion current diagram of quality control, **(B)** UPLC-QE ESI(−) total ion current diagram of quality control.

**FIGURE 5:**
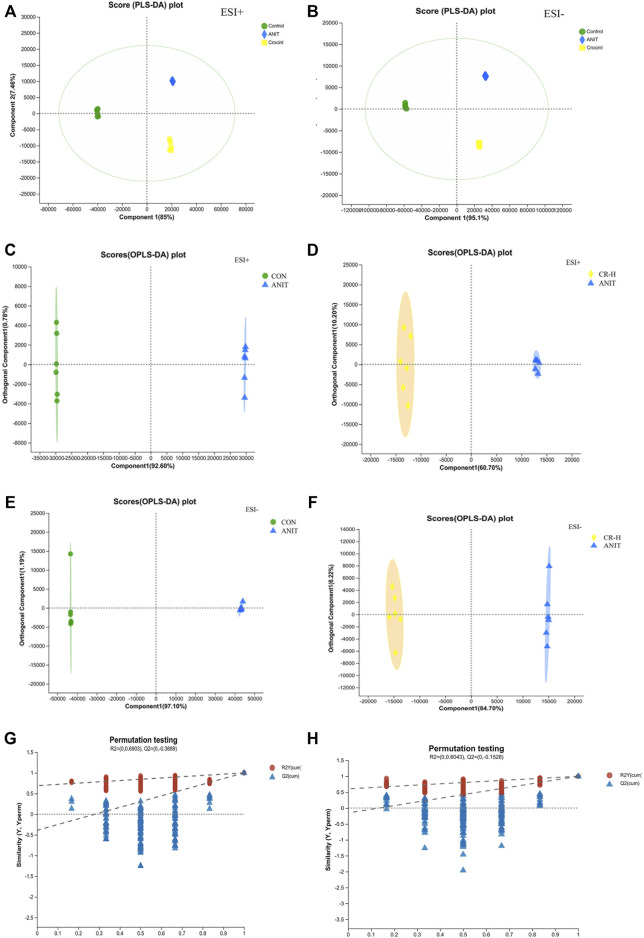
Multivariate statistical analysis of serum metabolomics. **(A),(B)** PLS-DA score plots of serum metabolomics analysis in the ESI + model and ESI− ANIT; **(C)** OPLS-DA score plots of CON and ANIT in positive mode; **(D)** OPLS-DA score plots of CON and CR-H in positive mode; **(E)** OPLS-DA score plots of CON and ANIT in negative mode; **(F)** OPLS-DA score plots of CON and CR-H in negative mode; **(G)** 200-permutation test of the OPLS-DA model for the CON and ANIT groups; **(H)** 200-permutation test of the OPLS-DA model for the CR and ANIT groups.

UPLC-Q Exactive HF-X/MS was used for the metabolite separation and data collection of serum samples. The serum metabolic profiles of rats in each group were obtained and indicated that some rat metabolites changed. Figure 4 shows typical chromatograms of positive and negative modes. As shown in Figures 5A,B, 85% and 95.1% of metabolites had a relative standard deviation (RSD)%≤30%, indicating that the separation among the different groups was good in the positive and negative ion modes, and the degree of polymerization within the group was high, indicating that there were remarkable differences in metabolites among the groups. The R^2^X (cum), R^2^Y (cum), and Q^2^ (cum) of OPLS-DA in our positive model were .926, 1, and 1, respectively, using the data from the CON and ANIT groups and .607, .993, and .986, respectively, using the data from the ANIT and CR groups (Figures 5C,D). The OPLS-DA was seen to be accurate and reliable. The R^2^X (cum), R^2^Y (cum), and Q^2^ (cum) of OPLS-DA in our negative model were .971, 1, and1, respectively, using the data from the CON and ANIT groups and .847, .993, and .992, respectively, using the data from the ANIT and CR groups (Figures 5E,F). The OPLS-DA was seen to be accurate and reliable. After 200 replacements, Figures 5G,H show values of *R* ^2^ > 0 and Q^2^ < 0, indicating that the model did not have overfitting, and that the OPLS-DA model is reliable.

### 3.3 Identification of potential metabolite of CR in the treatment of ANIT-induced IC

**FIGURE 6:**
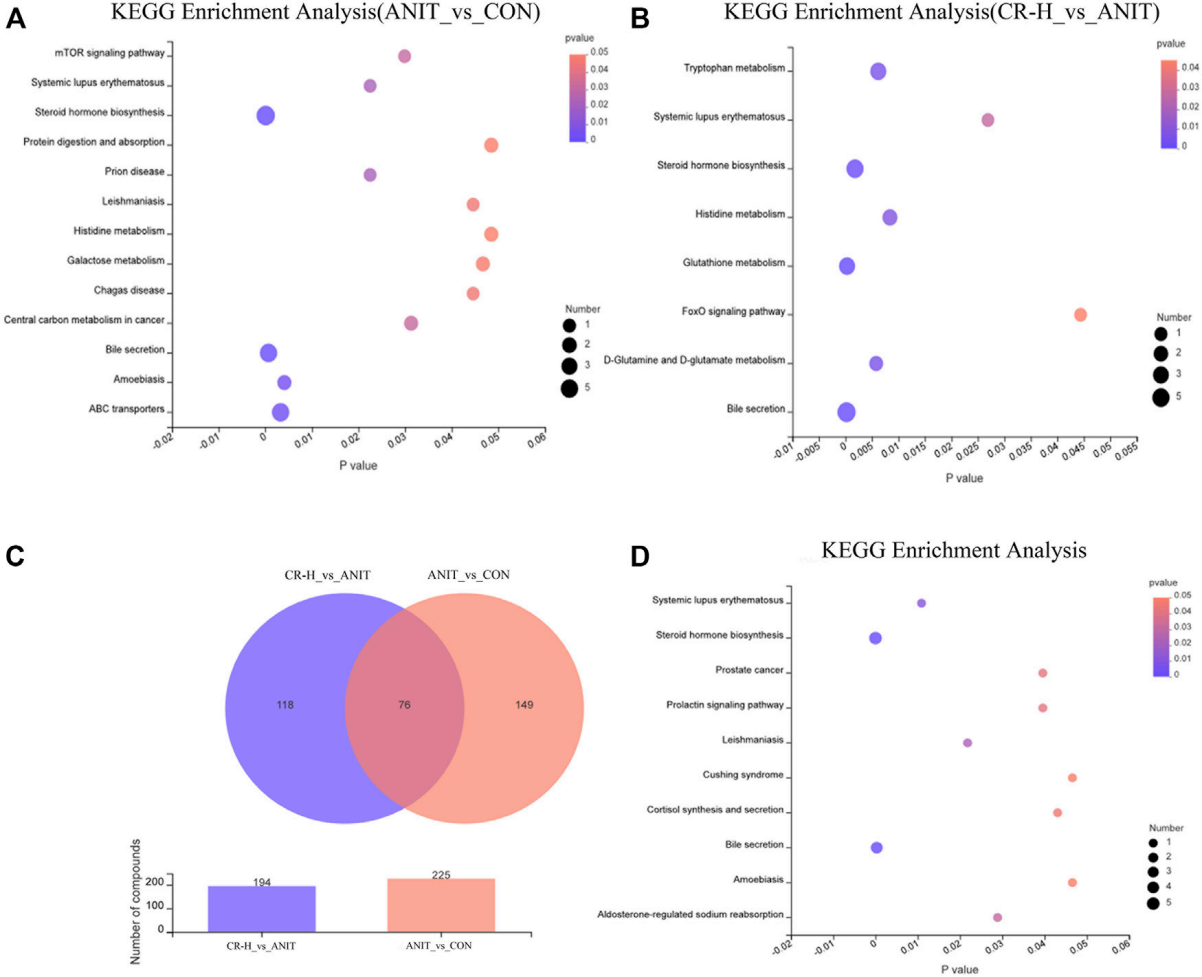
Metabolomics pathway enrichment analysis results. **(A, B)** KEGG pathway enrichment analysis of differential metabolites between the CON group, the ANIT group, and the CR group by bubble plot; **(C)** Venn diagram of differential metabolites between the groups; **(D)** pathway enrichment bubble map for 76 shared metabolites.

Based on Student’s t-test combined with the OPLS-DA analysis method, potential biomarkers were screened according to the conditions of VIP>1 and *p*-value < .05. A total of 225 significantly different metabolomics was identified between the ANIT group and the CON group, and 194 significantly different metabolites were obtained between the ANIT group and the CR-H group. To further reveal the metabolic pathways of potential metabolites related to CR in the treatment of IC, we analyzed the metabolic pathway using the KEGG database. Figure 6A shows the top 20 pathways involved in IC: three were significantly affected (*p* < .05), including bile secretion, steroid hormone biosynthesis, and ABC transporters.

The differential metabolites between the CR group and the ANIT group involved eight metabolic pathways, including bile secretion, glutathione metabolism, steroid hormone biosynthesis, d-glutamine and d-glutamate metabolism, tryptophan metabolism, histidine metabolism, systemic lupus erythematosus, and the foxo signaling pathway (Figure 6B). Venn analysis identified 76 metabolites as differential metabolites in the CR-treated IC rats (Figure 6C), which enriched 10 metabolic pathways (Figure 6D). Table 1 shows the identified intersected metabolites; these differential metabolites in serum may be potential target biomarkers for IC.

**TABLE 1:** Identification of potential biomarkers in serum.

Metabolite	Library id	Formula	tR/min	M/Z	Mode	VIP	*p*_value	Trend	
								M/K	CR/M
Naphthalene epoxide	HMDB0006215	C_10_H_8_O	5.36	186.091	pos	2.15	9.99E-17	↑**	↓**
Cervonoyl ethanolamide	HMDB0013627	C_24_H_36_O_3_	5.85	337.252	pos	1.08	1.95E-09	↑**	↓**
4,4′-Dihydroxy-5,5′-diisopropyl-2,2′-dimethyl-3,6-biphenyldione	HMDB0040760	C_20_H_24_O_4_	6.00	311.164	pos	4.79	5.93E-12	↑**	↑**
Dityrosine	HMDB0006045	C_18_H_20_N_2_O_6_	5.96	424.150	pos	1.07	6.38E-06	↑**	—
7-beta-D-Glucopyranosyloxybutylidenephthalide	HMDB0034752	C_18_H_22_O_8_	5.26	367.140	pos	1.12	6.97E-07	↑**	—
5′-Carboxy-gamma-chromanol	HMDB0012799	C_18_H_26_O_4_	.51	345.148	pos	1.34	3.29E-09	↑**	↓**
Quillaic acid 3-[xylosyl-(1->3)-[galactosyl-(1->2)]-glucuronide]	HMDB0033406	C_47_H_72_O_20_	2.05	490.232	pos	1.00	8.95E-07	↓**	↑**
4-(3-Pyridyl)-3-butenoic acid	HMDB0001424	C_9_H_9_NO_2_	2.57	164.071	pos	1.28	2.83E-08	↓**	↑**
Estrone	HMDB0000145	C_18_H_22_O_2_	3.33	315.134	pos	1.46	.001057	↑**	↑**
Bilirubin	HMDB0000054	C_33_H_36_N_4_O_6_	5.88	585.270	pos	1.17	.001869	↑**	↓**
Matricin	HMDB0036643	C_17_H_22_O_5_	5.92	613.302	pos	1.33	1.20E-05	↑**	↓**
Dehydroabietic acid	HMDB0061925	C_20_H_28_O_2_	6.08	301.216	pos	2.29	1.76E-11	↑**	↓**
2-Amino-14,16-dimethyloctadecan-3-ol	LMSP01080031	C_20_H_43_NO	6.11	314.341	pos	1.09	4.35E-05	↓**	↑**
PS (18:0/20:4 (8Z,11Z,14Z,17Z))	HMDB0010165	C_44_H_78_NO_10_P	6.55	812.541	pos	1.57	5.36E-06	↑**	↓**
Suspensolide F	HMDB0031918	C_21_H_34_O_12_	6.70	501.196	pos	1.26	1.27E-06	↓**	↑**
Lithocholyltaurine	HMDB0000722	C_26_H_45_NO_5_S	8.41	466.298	pos	1.55	1.49E-08	↑**	↓**
Cucurbitacin D	HMDB0034695	C_30_H_44_O_7_	7.33	580.327	pos	1.90	1.36E-05	↑**	↓**
PE-NMe2 (20:2 (11Z,14Z)/18:4 (6Z,9Z,12Z,15Z))	HMDB0114250	C_45_H_78_NO_8_P	6.96	814.536	pos	1.51	4.45E-05	↓**	↑**
PE-NMe2 (11D3/11M5)	HMDB0114663	C_48_H_84_NO_10_P	6.68	848.576	pos	2.06	2.96E-08	↑**	↓**
6-Hydroxyshogaol	HMDB0041249	C_17_H_24_O_4_	6.16	331.130	pos	1.02	7.54E-06	↑**	↓**
Gamma-Glutaminyl-4-hydroxybenzene	HMDB0029451	C_11_H_14_N_2_O_4_	6.15	494.228	pos	1.66	.0001696	↑**	↓**
LysoPC (10:0)	HMDB0003752	C_18_H_39_NO_7_P+	6.14	413.254	pos	1.40	8.94E-07	↑**	↓**
Physalin O	HMDB0039081	C_28_H_32_O_10_	6.11	529.208	pos	1.08	9.38E-06	↑**	—
C17 Sphinganine	LMSP01040003	C_17_H_37_NO_2_	6.01	288.289	pos	1.13	1.99E-09	↓**	↑**
Fumonisin B2	HMDB0034703	C_34_H_59_NO_14_	5.94	670.379	pos	1.88	9.94E-11	↑**	↑**
Cortisol	HMDB0000063	C_21_H_30_O_5_	5.88	345.206	pos	1.02	.006046	↓**	↑**
Tauroursodeoxycholic acid	HMDB0000874	C_26_H_45_NO_6_S	5.69	464.283	pos	1.21	1.38E-10	↑**	—
Isoleucyl-methionine	HMDB0028913	C_11_H_22_N_2_O_3_S	5.62	301.100	pos	1.18	8.57E-09	↑**	↓**
31-Hydroxy rifabutin	HMDB0060754	C_46_H_62_N_4_O_12_	5.40	904.477	pos	1.96	6.93E-06	↓**	↑**
Cysteinylglycine	HMDB0000078	C_5_H_10_N_2_O_3_S	5.34	242.058	pos	2.12	.000165	↓**	↑**
Glutathione episulfonium ion	HMDB0060479	C_12_H_20_N_3_O_6_S^+^	5.02	367.140	pos	1.08	5.89E-06	↑**	↓**
(9Z,11E,13E,15Z)-4-Oxo-9,11,13,15-octadecatetraenoic acid	HMDB0031098	C_18_H_26_O_3_	4.49	329.149	pos	1.04	2.79E-06	↑**	—
Isoleucyl-cysteine	HMDB0028904	C_9_H_18_N_2_O_3_S	4.06	273.069	pos	1.94	4.32E-13	↑**	↓**
Myricanone	HMDB0030798	C_21_H_24_O_5_	3.73	379.150	pos	1.64	7.88E-10	↑**	↓**
L,L-Cyclo (leucylprolyl)	HMDB0034276	C_11_H_18_N_2_O_2_	3.70	211.144	pos	1.06	1.23E-05	↑**	↓**
Isoquinoline	HMDB0034244	C_9_H_7_N	3.70	130.065	pos	1.20	4.91E-10	↑**	↓**
N-Deschlorobenzoyl indomethacin	HMDB0013988	C_12_H_13_NO_3_	2.45	202.086	pos	1.33	2.39E-11	↑**	—
N-(1-Deoxy-1-fructosyl)phenylalanine	HMDB0037846	C_15_H_21_NO_7_	2.21	328.139	pos	1.43	1.06E-07	↓**	↑**
6-(4-Ethyl-2-hydroxyphenoxy)-3,4,5-trihydroxyoxane-2-carboxylic acid	HMDB0124984	C_14_H_18_O_8_	1.89	356.134	pos	1.46	1.52E-07	↑**	↓**
Niazirinin	HMDB0032808	C_16_H_19_NO_6_	1.47	339.155	pos	2.26	8.69E-09	↓**	↑**
Isovalerylalanine	HMDB0000747	C_8_H_15_NO_3_	.99	174.112	pos	1.28	1.79E-06	↓**	↑**
1-[(5-Amino-5-carboxypentyl)amino]-1-deoxyfructose	HMDB0034879	C12H24N2O7	.51	353.129	pos	1.32	3.71E-06	↓**	↑**
Carnosine	HMDB0000033	C9H14N4O3	.50	227.114	pos	1.35	2.25E-10	↓**	↑**
Taurolithocholic acid 3-sulfate	HMDB0002580	C26H45NO8S2	6.02	280.621	neg	1.22	2.07E-13	↑**	—
12-Ketodeoxycholic acid	HMDB0000328	C24H38O4	6.15	435.275	neg	1.12	.001512	↓**	↑**
4-Chloro-1H-indole-3-acetic acid	HMDB0032936	C10H8ClNO2	6.00	208.016	neg	1.08	2.14E-07	↓**	↑**
2-trans-4-trans-7-cis-Decatrienal	HMDB0032213	C10H14O	4.49	195.102	neg	1.51	.02487	↓**	↓*
Pinocembrin	LMPK12140214	C15H12O4	5.76	255.066	neg	5.85	4.39E-10	↑**	↓**
Taurochenodeoxycholate-7-sulfate	HMDB0002498	C26H45NO9S2	5.92	560.234	neg	1.20	1.42E-07	↑**	—
Rac-5,6-Epoxy-retinoyl-beta-D-glucuronide	HMDB0060123	C26H36O9	6.04	491.228	neg	1.93	1.35E-14	↑**	↓**
Protoporphyrinogen IX	HMDB0001097	C34H40N4O4	6.11	567.297	neg	1.35	1.59E-07	↑**	↑**
1,11-Undecanedicarboxylic acid	HMDB0002327	C13H24O4	6.12	243.160	neg	1.89	7.54E-06	↓**	↑**
CPA (18:2 (9Z,12Z)/0:0)	HMDB0007007	C21H37O6P	8.11	415.225	neg	2.34	4.05E-13	↓**	↑**
Ganoderic acid eta	HMDB0036309	C30H44O8	6.78	553.283	neg	1.61	2.07E-09	↑**	—
Frangulanine	HMDB0030199	C28H44N4O4	6.70	537.288	neg	1.44	9.63E-07	↑**	—
Androsterone glucuronide	HMDB0002829	C25H38O8	6.70	501.222	neg	1.07	1.71E-06	↓**	↑**
Janthitrem C	HMDB0040684	C37H47NO4	6.14	604.325	neg	2.25	.0001456	↓**	↑**
3-Hydroxytetradecanedioic acid	HMDB0000394	C14H26O5	6.12	255.160	neg	1.54	2.55E-06	↓**	↑**
Allixin	HMDB0040705	C12H18O4	6.08	207.102	neg	1.01	4.04E-06	↓**	↑**
Kinetin-7-N-glucoside	HMDB0012243	C16H19N5O6	5.95	422.135	neg	1.36	1.28E-08	↑**	↑**
Ganglioside GT3 (d18:1/20:0)	HMDB0012073	C83H146N4O37	5.79	894.469	neg	2.85	8.92E-10	↓**	↑**
Melleolide	HMDB0035689	C23H28O6	5.55	445.189	neg	1.31	3.37E-11	↑**	↑**
Imazamethabenz	HMDB0034885	C15H18N2O3	3.57	255.113	neg	2.01	4.13E-09	↑**	↑**
5-Hydroxyindoleacetic acid	HMDB0000763	C10H9NO3	3.49	190.050	neg	1.05	1.04E-07	↓**	↓**

Note: M/K: ANIT versus CON,CR/M:CR-H versus ANIT; ↑:content increased,↓:content decreased; *p < .05,**p < .01.

### 3.4 Transcriptomic data analysis

**FIGURE 7:**
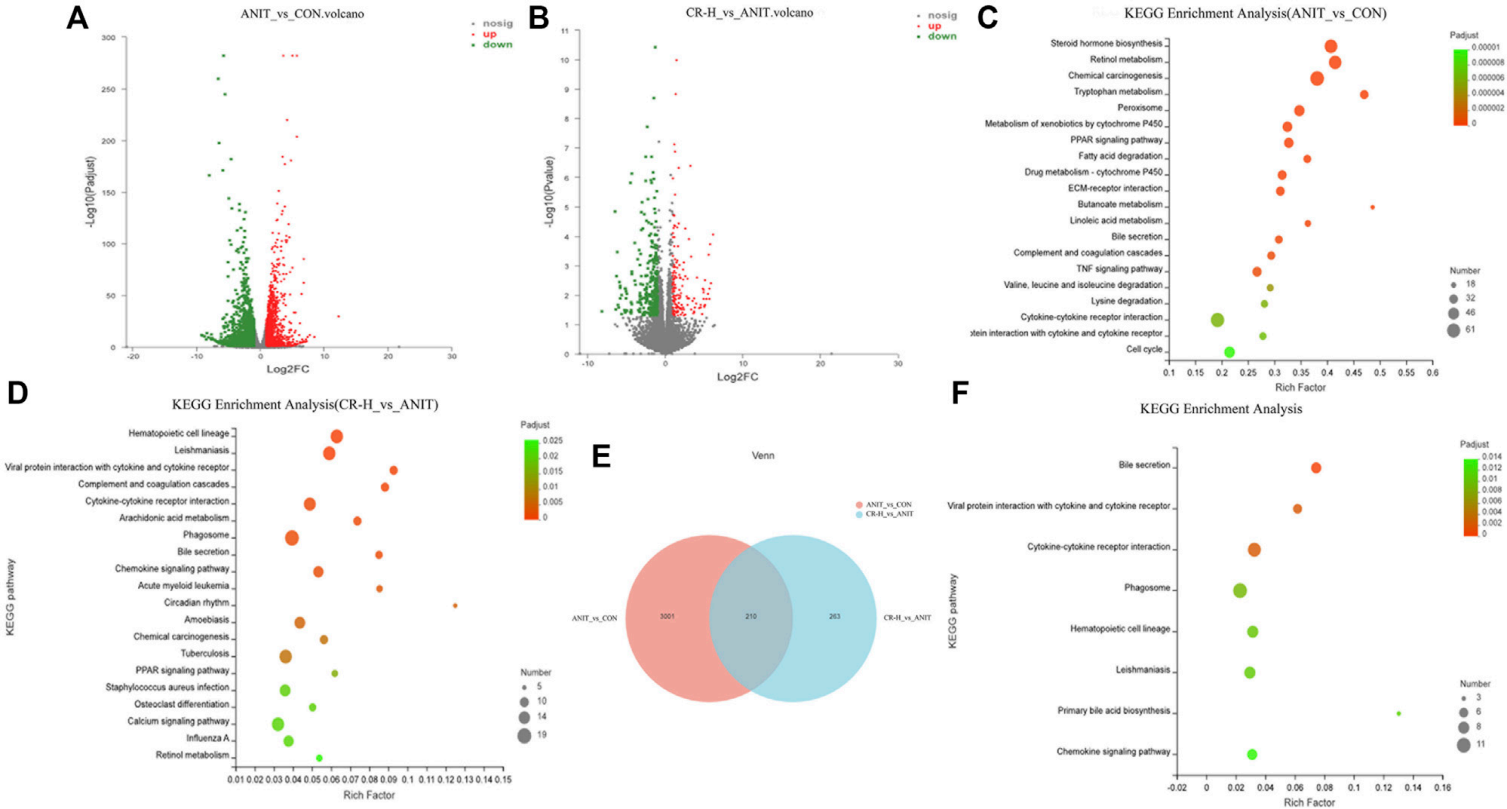
Transcriptomic data analysis (CON, ANIT, and CR-H groups; n = 3). **(A, B)** Volcano map of between-group DEGs; **(C)** the KEGG pathway analysis of the ANIT and CON groups; **(D)** the KEGG pathway analysis of the CR-H and ANIT groups; **(E)** the Venn diagram of DEGs among groups; **(F)** the KEGG pathway analysis of 210 DEGs.

The DEGs were screened using the software DEseq2, with |log2(Fold Change) |>1 and *p*-value_adjust<.05 as the screening conditions. Compared ANIT group with the CON group, 1806 DEGs were significantly upregulated, and 1405 DEGs were significantly downregulated (Figure 7A). Compared with the ANIT group, 153 genes were upregulated, and 320 genes were downregulated in the CR-H group (Figure 7B). With *p*-value_adjust<.05 as the filter condition, the DEGs between the ANIT group and the CON group were mainly enriched in tryptophan metabolism, chemical carcinogenesis, retinol metabolism, steroid hormone biosynthesis, cell cycle, bile secretion, linoleic acid metabolism, butanoate metabolism, ECM-receptor interaction, drug metabolism-cytochrome P450, fatty acid degradation, PPAR signaling pathway, and the TNF signaling pathway (Figure 7C). These findings show that the pathways that cause cholestasis are closely related to these factors. With *p*-value_adjust<.05 as the filter condition, the DEGs between the CR group and the ANIT group were mainly enriched in the retinol metabolism, calcium signal pathway, PPAR signaling pathway, circadian rhythm, chemokine signal pathway, arachidonic acid metabolism, bile secretion, and primary bile acid biosynthesis (Figure 7D). A total of 3,211 DEGs were obtained between the CON group and the ANIT group, and 210 DEGs were regulated after administration (Figure 7E). Figure 7F shows the KEGG pathway enriched by 210 DEGs.

### 3.5 Integrated analysis of transcriptomics and metabolomics

**FIGURE 8:**
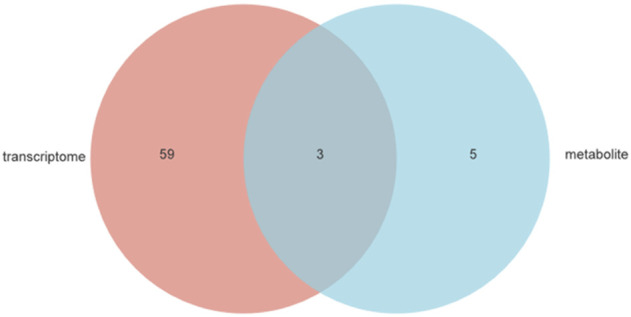
Venn diagram: Pathways with significant enrichment of genes and metabolites.

Venn analysis was performed to reflect the common pathways annotated between the DEGs of the transcriptome and the differential metabolites of the metabolomics, and three shared pathways were obtained, including steroid hormone biosynthesis, systemic lupus erythematosus, and bile secretion (Figure 8). With *p*-value_adjust<.05 as the filter condition, one pathway was significantly enriched: bile secretion (Table 2).

**TABLE 2:** KEGG shared pathway data table.

Pathway id	Pathway description	First category	Second category	Metabolite list	Gene list	*p*-value_ corrected
rno00140	Steroid hormone biosynthesis	Metabolism	Lipid metabolism	Androsterone glucuronide; cortisol; estrone	gene29387(Ugt2b7); gene1452(Cyp2b2)	.1571
rno05322	Systemic lupus erythematosus	Human diseases	Immune disease	PS (18:0/20:4 (8Z,11Z,14Z,17Z))	gene36403 (RT1-Db1); gene36402 (RT1-Ba	.0529
					gene36406 (RT1-Da); gene36267(C4a)	
					gene36497 (Tnf); gene28554(Fcgr3a)	
rno04976	Bile secretion	Organismal systems	Digestive system	6-Hydroxy-5-methoxyindole glucuronide; bilirubin; 5-hydroxy-6-methoxyindole glucuronide; estrone; cortisol; glycocholic acid; taurolithocholic acid 3-sulfate	gene13714(Acnat1); gene1200(Sult2a1); gene1197(Sult2a2); gene15392 (Abcg8); gene24758 (Abcc3); gene15391 (ABCG5); gene5199(HMGCR)	.0015

### 3.6 Expression correlation analysis

**FIGURE 9:**
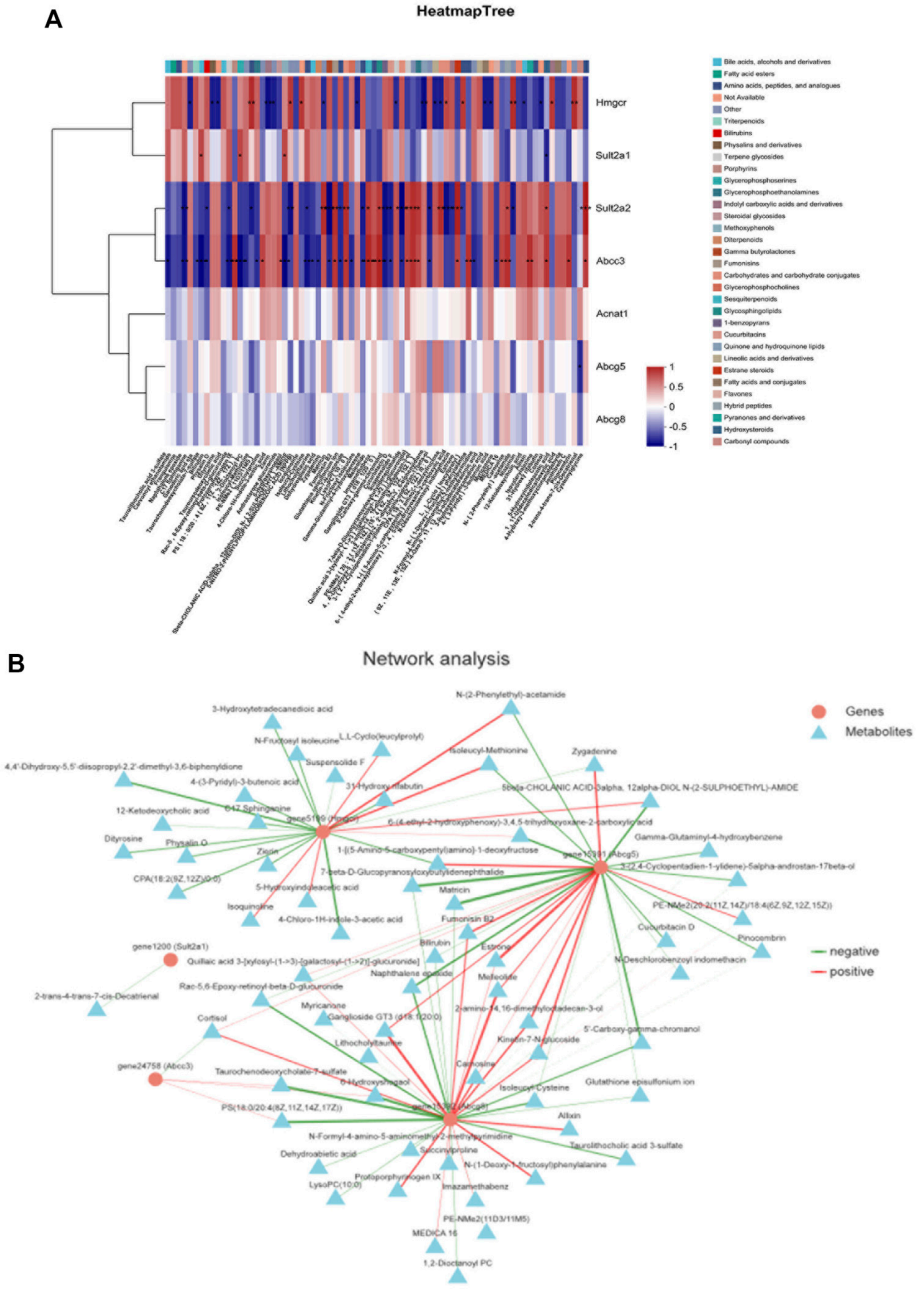
Correlation analysis of metabolic biomarkers and DEGs based on Spearman rank. **(A)** Correlation heat map, **(B)** correlation network map.

Spearman rank correlation was applied to analyze the DEGs and metabolites. Corr values greater than 0 identify a positive correlation between the differential metabolite and the differential gene. Corr values less than 0 identify a negative correlation between the differential metabolite and the differential gene. The redder the color, the stronger the positive correlation, and the bluer the color, the stronger the negative correlation. A Corr value of 0 indicates no correlation. The correlation between HMGCR, SULT2A1, Sult2a2, Abcc3, and metabolites was strong, and Acnat1, ABCG5, and Abcg8 had a potential correlation with some metabolites (Figure 9A).

The DEGs and differential metabolites on key pathways were imported into Cytoscape 3.8.2 software for gene-metabolite interaction network analysis. The interaction relationship between differential metabolites and DEGs was displayed using the network diagram. As shown in Figure 9B, the relationship between genes and metabolites was established. The results showed that the HMGCR, ABCG5, and Abcg8 genes interacted with most metabolites, while the Abcc3 and SULT2A1 genes interacted with a few metabolites.

### 3.7 The effect of CR on the expression of key targets

**FIGURE 10:**
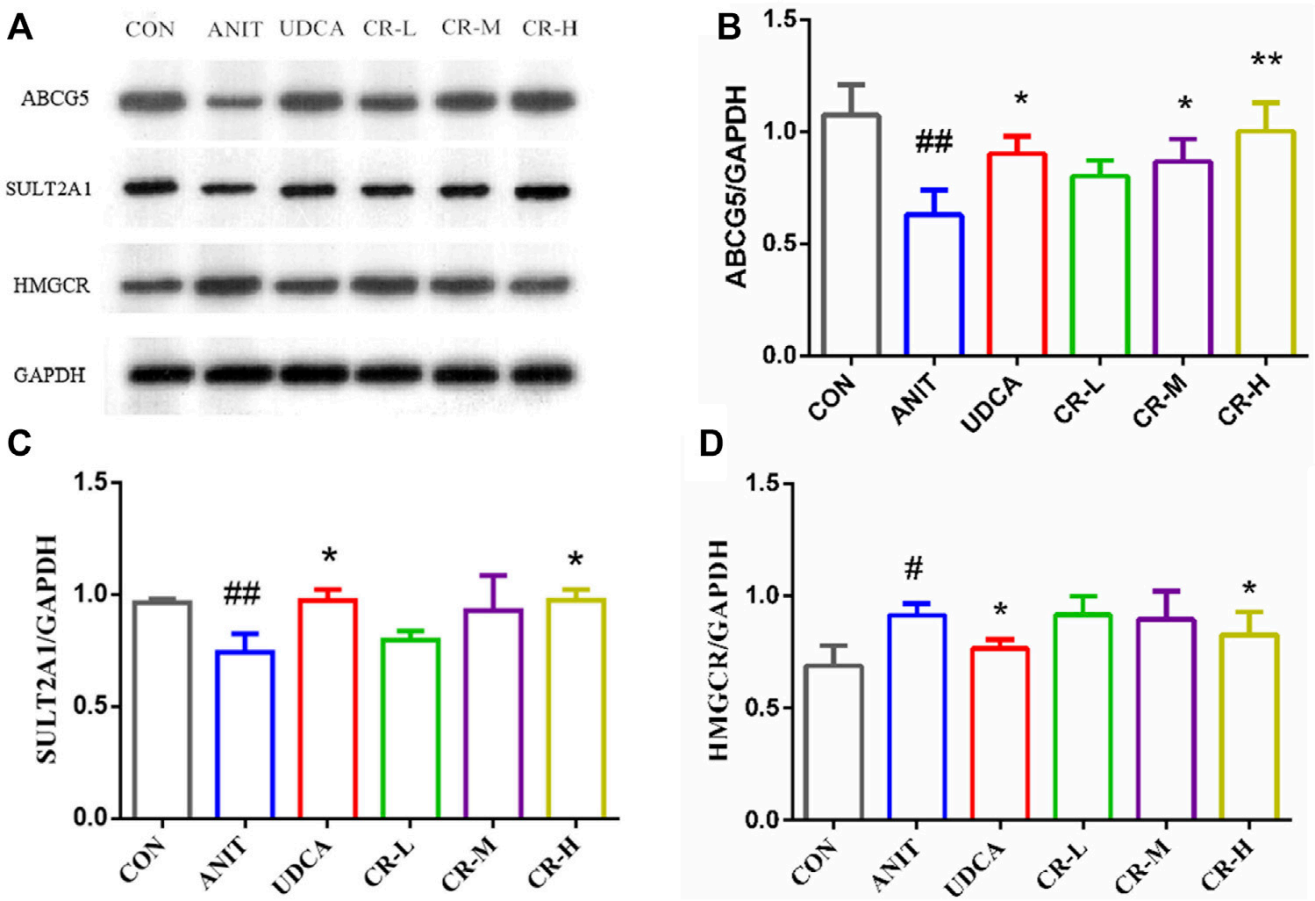
Expression levels of different proteins in the liver tissue of rats after administration. **(A)** Electrophoretic diagram of different groups, **(B)** expression level of ABCG5 protein, **(C)** expression level of SULT2A1 protein, **(D)** expression level of HMGCR protein.

We used immunoblotting to validate the targets that directly modulate potential metabolites and verify some differential genes in the bile secretion pathway. The results showed that the expression of HMGCR in the liver tissue of rats in the ANIT group was significantly increased compared with the CON group. The expressions of ABCG5 and SULT2A1 levels were significantly decreased, and the expressions of these proteins were significantly regulated in CR (*p* < .01 or *p* < .05, Figure 10).

## 4 Discussion

Metabolomics is a qualitative or quantitative analysis of all small molecule metabolites in the organism with a relative molecular mass between 50 and 1500 to find the links between metabolites and physiological/pathological changes (Zhao et al., 2014). We identified 76 significant metabolites of the CR group in the serum, as well as their related pathways. In view of the one-sidedness of single omics, data analysis and interpretation were performed using transcriptomic conjoint analysis methods. We found three key targets (ABCG5, HMGCR, SULT2A1) and one pathway (bile secretion). Integration of metabolomics and transcriptomics was designed to study the metabolite profiles and gene expression profiles in biological systems under administration conditions and analyze the internal changes of biological systems from two levels (Chen et al., 2020).

In this study, we investigated the mechanism of the effect of CR against IC through metabolomics and transcriptomics. The IC model was successfully established by the administration of ANIT olive oil solution. Compared with the CON group, the serum levels of AST, TBA, and TBIL in the ANIT group were significantly increased, while they were significantly inhibited in the CR groups, which was consistent with the result of Xiong et al. (2016). In addition, compared with the ANIT group, the levels of TNF-α, 1L-Lβ, and 1L-6 in serum were significantly inhibited after CR administration, indicating that CR could play a protective and therapeutic role by alleviating inflammation, which was consistent with histological analysis.

Cholestasis is closely related to metabolic disorders. Metabolomics can reveal the changes in the spectrum of endogenous metabolites in the body and further analyze the overall biological status and functional regulation of the body (S. Chen et al., 2022; Lin et al., 2020). After CR treatment, 45 metabolites in the serum were regulated. These candidate biomarkers and pathways suggest that the pathogenesis of cholestasis disease is a complex process. Therefore, the transcriptomic analysis was applied to explore the mechanism of CR when treating IC. In our study, 3,211 DEGs were obtained between the CON and the ANIT group. Compared with the ANIT group, 148 genes were regulated in the CR group. In addition, the enrichment pathways were mainly involved with endocrine system-related pathways, signal transduction pathways, and lipid metabolism pathways*.* Using an integrated strategy of transcriptomic and metabolomic analysis revealed the mechanism of CR in the treatment of IC.

To further understand the regulation of potential biomarkers in the CR group, we used the Spearman correlation algorithm to perform correlation analysis on DEGs and differentially expressed metabolites. The targets and potential biomarkers of the CR group were analyzed, and an interactive network of “potential-biomarker-target-components” was constructed. The results showed that the bile secretion pathway was the most important pathway in IC, and the most closely potential biomarkers were HMGCR, SULT2A1, ABCG5, ABCG8, and ABCC3.

The increase of bilirubin is one of the important phenomena of IC. Direct bilirubin is produced by indirect bilirubin through the transformation of bilirubin in the blood by hepatocytes in the liver (Kong et al., 2021). Further correlation analysis of DEGs and DEMs revealed that SULT2A1, HMGCR, are positively correlated with bilirubin, and ABCG5 are negatively correlated with bilirubin. The corresponding proteins of these three genes were verified by western blot. The analysis of expression of ABCG5, SULT2A1, and HMGCR was consistent with the western blot results, indicating that the results at the gene level and protein level were consistent, and the results of the bioinformatics analysis were reliable.

Synthetic and metabolic enzymes of bile acids also play significant roles in bile acid homeostasis. SULT2A1 can play an important role in the bile secretion pathway (Wu et al., 2021). This study found that the expression level of SULT2A1 in the ANIT group was significantly lower than that in the CON group. The expression of SULT2A1 can be significantly increased after the high-dose intervention of CR, indicating that CR at a dose of 90 mg/kg can exert a detoxification effect by regulating bile acid metabolism. Cholestasis can also lead to abnormal cholesterol metabolism. The liver cholesterol transporter ABCG5/8 is a member of the ABC transporter family, and it is highly expressed in the liver. In this study, the expression of ABCG5/8 in the ANIT group was lower than that of the CON group, and cholestasis developed. In the CR group, the expression of ABCG5/8 could be reversed, and the liver injury was relieved. Multidrug resistance-related protein 3 (MRP3/ABCC3) belongs to the ABC transporter superfamily, which can combine ATP and utilize energy to drive a variety of different molecules across cell membranes (Wang et al., 2021). MRP3 is responsible for the transfer of certain specific bile acids, and the part of MRP3 may be compensated for by other transporters (Perez-Pineda et al., 2021). In this study, the expression level of HMGCR protein in the ANIT group was increased. In the CR-H group, this protein was significantly downregulated, indicating that the cholesterol synthesis ability was inhibited, and the secretion of bile acids was relatively reduced.

The concentration of 12-ketodeoxycholic acid in the bile of rats in the ANIT group was significantly higher than that in the control group, suggesting that bile acid metabolism in rats was significantly interfered with, thus causing lipid metabolism dysfunction. Due to liver function injury, bilirubin secretion and synthesis of primary bile acid in liver cells increased, which promoted the excretion of 12-ketodeoxycholic acid and increased the detected amount of 12-ketodeoxycholic acid in bile. In the ANIT group, the levels of sulfated cholate in the liver and serum were significantly increased, suggesting that the body had a compensatory detoxification process and alleviated the hepatocyte accumulation toxicity of cholate.

## 5 Conclusion

In this study, a novel integrated method was designed to investigate the key targets and mechanisms of CR when treating IC according to metabolomics and transcriptomics. The current results showed that CR has the effect of improving IC. The integrated analysis revealed three key targets as well as related metabolites and pathways. The targets were further verified by western blotting. This study has provided data and theoretical support for an in-depth study of the mechanism and has laid the foundation for clinical application. Further systematic molecular biology experiments are needed to verify the accurate mechanisms. The study has also provided a novel paradigm to identify the potential mechanisms of pharmacological effects derived from a natural compound.

## Data Availability

The raw data supporting the conclusions of this article will be made available by the authors without undue reservation.
